# Identification of androgen-responsive lncRNAs as diagnostic and prognostic markers for prostate cancer

**DOI:** 10.18632/oncotarget.11391

**Published:** 2016-08-19

**Authors:** Xuechao Wan, Wenhua Huang, Shu Yang, Yalong Zhang, Honglei Pu, Fangqiu Fu, Yan Huang, Hai Wu, Tao Li, Yao Li

**Affiliations:** ^1^ State Key Laboratory of Genetic Engineering, Shanghai Engineering Research Center Of Industrial Microorganisms, School of Life Science, Fudan University, Shanghai 200433, PR China; ^2^ Key Laboratory of Reproduction Regulation of NPFPC, Fudan University, Shanghai 200433, PR China; ^3^ Department of Urology, Tongji Hospital, Tongji University School of Medicine, Shanghai 200433, PR China

**Keywords:** long non-coding RNA, prostate cancer, androgen receptor, expression profiling, biomarkers

## Abstract

Prostate cancer (PCa) is a leading cause of mortality among males. Long non-coding RNAs (lncRNAs) are subclass of noncoding RNAs that may act as biomarkers and therapeutic targets. In this study, we firstly conducted analysis of global lncRNA expression patterns by using our own cohort (GSE73397) and two public available gene expression datasets: The Cancer Genome Atlas (TCGA) and GSE55909. Next, we performed microarray to observe genome-wide lncRNAs' expressions under dihydrotestosterone (DHT) stimulation in LNCaP cells (GSE72866), and overlapped the result with ChIPBase data to predict androgen-responsive lncRNAs with ARE. Combined the two results, a total of 44 androgen-responsive lncRNAs with ARE were found to be over-expressed in PCa samples. Ten lncRNAs were selected for further validation by examining their expressions in LNCaP cells under DHT stimulation, and in PCa samples and cell lines. Among them, RP1-4514.2, LINC01138, SUZ12P1 and KLKP1 were validated as directly AR-targeted lncRNAs by ChIP-PCR. Then we conducted a bioinformatic analysis to identify lncRNAs as putative prognostic and therapeutic targets by using TCGA data. Three androgen-responsive lncRNAs, LINC01138, SUZ12P1 and SNHG1 showed association with gleason score and pT-stage. The biological functions of LINC01138 and SUZ12P1 were also evaluated, both lncRNAs promoted the proliferation and inhibited apoptosis of PCa. These results provide potent information for exploring potential biomarkers and therapeutic targets for prostate cancer, especially for castration-resistant PCa.

## INTRODUCTION

Long noncoding RNAs (lncRNAs) are RNA polymerase II (RNAPII) transcripts of more than 200 nucleotides with no protein coding function [1]. Increasingly reports show that lncRNAs play important roles in tumorigenesis, cancer progression, and metastasis by regulating expression of protein-coding genes through transcriptional, post-transcriptional, post-translational and/or epigenetic regulation [2–5]. For example, lncRNA CTBP1-AS, associates with PSF, recruits the HDAC–Sin3A complexes to CTBP1 promoter in *cis* and also guides PSF complexes to the regulatory regions of their endogenous target genes in *trans*-regulatory pathway [6]. LncRNAs play different roles in various cancers, either oncogenes (PCGEM1, CTBP1-AS) or tumor suppressor genes (GAS5, LincRNA-p21 and PCAT29) [7–10].

Prostate cancer (PCa) is the most commonly diagnosed cancer of men in the United States with secondly leading cause of death in 2015 [11]. Androgen receptor (AR) play a pivotal role in prostate cancer (PCa) development at all stages, including the “androgen-independent” tumors [12]. Androgen ablation is a primary therapy of prostate cancer. However, after 18 to 24 months of androgen deprivation, PCa will eventually progress to castration-resistant form with limited therapeutic options and poor prognosis [13–15]. The accurate molecular mechanisms for hormone independent behavior of PCa remain unclear. Recently, emerging evidence suggests a few AR-regulated lncRNAs including CTBP1-AS, PCAT18 and PCAT29 show association with PCa androgen-resistance transformation [6, 16, 17]. Thus, we attempted to systematically identify androgen-responsive lncRNAs that may mediate the processes from “androgen-dependent” to “androgen-independent” in PCa.

On the other hand, risk stratification of PCa patients at diagnosis is mainly dependent on prostate-specific antigen (PSA) testing, which has low specificity for cancer distinguishing from PBH, and cannot discriminate between indolent and metastatic castration-resistant PCa (mCRPC) [18, 19]. Therefore, there is a significant need to identify potential novel prognostic biomarkers. Several previously characterized lncRNAs (SChLAP1, PCGEM1 and PCA3) show disease-associated overexpression [20–22]. Notably, PCA3 showed better performance than PSA in urinary detection to detect PCa [23]. Unfortunately, PCA3 still couldn't be able to discriminate between indolent and clinically aggressive PCa [24].

In this study, we firstly conducted analysis of global lncRNA expression patterns by using our own cohort (GSE73397) and two public available gene expression datasets: The Cancer Genome Atlas (TCGA) and GSE55909. Next, we performed microarray to observe genome-wide lncRNAs' expressions under dihydrotestosterone (DHT) stimulation in LNCaP cells (GSE72866). Then we conduct a bioinformatic analysis to identify lncRNAs as putative prognostic and therapeutic targets by using TCGA data. Our results suggest that the lncRNAs we report may provide new molecular biomarkers for the diagnosis of prostate cancer, especially for castration-resistant PCa.

## RESULTS

### Identification of differentially expressed androgen-responsive lncRNAs in prostate cancer patients

**Figure 1:**
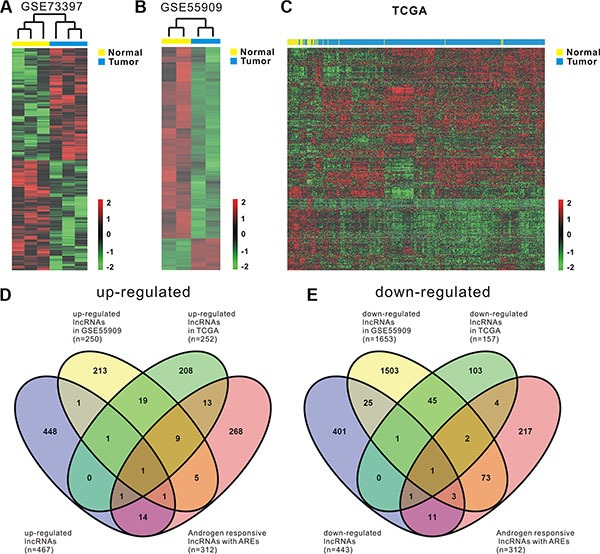
Comprehensive analysis of differentially expressed androgen-responsive lncRNAs in PCa patients Heat map shows differential lncRNA expression between prostate tumor samples and normal tissues by using our own cohort (GSE73397) (**A**) and two publicly available gene expression data GSE55909 (**B**) and TCGA (**C**). Hierarchical clustering reveals distinguishable lncRNA expression profiles. Red indicates high relative expression and green indicates low relative expression. 2, 0 and -2 are folds changes in the corresponding spectrum, whereas Normal represents normal prostate samples and Tumor represents prostate cancer tissues. (**D**, **E**) four-way Venn diagrams display the overlap of up-regulated and down-regulated androgen-responsive lncRNAs that are in GSE73397, GSE55909, TCGA and GSE72866.

Global expressions of lncRNAs in PCa samples and normal prostate tissues were examined by RiboArray^™^Custom Array (Arraystar, Rockville, MD) and 982 lncRNAs with differential expression (*p* < 0.1) between PCa samples and normal prostate tissues were selected for further analysis. Of the lncRNAs, 501 lncRNA transcripts produced from 467 genes were up-regulated and 481 transcripts produced from 443 genes were down-regulated in prostate cancer. The list of the top 30 differentially expressed lncRNAs identified by microarray analysis was shown in Table 1. Hierarchical clustering of the differentially expressed lncRNAs was shown in Figure 1A.

**Table 1:** Top 30 differentially expressed lncRNAs in prostate cancer samples identified by microarray analysis

ProbeName	Gene Symbol	FC (abs)	pval	Regulation	T1	T2	T3	N1	N2	N3	Chr
ENST00000584428	RP5-890E16.2	3.92	0.04	down	10.74	11.07	9.16	11.82	12.87	12.19	Chr 17
ENST00000572222	MMP25-AS1	3.32	0.05	down	9.5	10.81	8.96	11.74	10.94	11.78	Chr 16
ENST00000425192	AC073641.2	3.09	0.02	down	8.52	7.82	7.57	8.97	10.17	9.66	Chr 2
ENST00000582858	RP11-192H23.8	2.64	0.04	down	9.88	10.18	9.51	11.95	11.39	10.43	Chr 17
ENST00000517345	RP11-30J20.1	2.48	0.06	down	8.78	7.63	7.68	8.65	9.61	9.75	Chr 8
ENST00000432195	RP4-575N6.4	2.34	0.04	down	7.68	8.34	7.1	9.18	9.04	8.57	Chr 1
ENST00000414668	LINC00029	2.3	0.1	down	7.96	9.33	9.48	9.62	10.61	10.16	Chr 20
ENST00000537370	CCND2-AS2	2.3	0.07	up	9.11	10.55	9.11	8.11	8.59	8.46	Chr 12
ENST00000523445	TPT1-AS1	2.25	0.07	down	8.03	9.41	8.03	9.42	9.92	9.65	Chr 13
ENST00000491849	LINC00971	2.19	0.03	up	9.58	10.49	9.86	8.57	8.69	9.28	Chr 3
ENST00000366437	MIR205HG	2.19	0.02	down	9.1	9.33	8.68	9.8	10.52	10.18	Chr 1
ENST00000443892	RP3-395M20.8	2.12	0.01	down	10.14	10.26	10.15	11.66	11.38	10.78	Chr 1
ENST00000530223	RP5-1047A19.4	2.07	0.08	up	11.41	10.29	9.99	9.38	9.49	9.67	Chr 8
ENST00000577848	RP11-874J12.4	2.01	0.05	down	8.8	9.4	8.5	9.49	9.87	10.36	Chr 18
ENST00000426364	LINC00633	2.01	0.03	up	10.46	9.45	9.88	8.9	9	8.88	Chr X
ENST00000577560	CTD-2006K23.1	1.98	0.1	down	8.48	9.03	7.89	10.09	9.26	9.01	Chr 17
ENST00000519967	RP11-705O24.1	1.97	0.09	up	10.4	9.93	10.05	8.87	8.64	9.95	Chr 8
ENST00000568635	CTA-363E6.5	1.95	0.04	up	9.6	10.32	9.64	8.88	8.51	9.29	Chr 16
ENST00000455238	RP11-193P11.3	1.94	0.04	down	9.34	9.82	8.69	10.19	10.3	10.22	Chr 1
ENST00000555070	RP11-33N16.3	1.94	0.01	up	9.54	10.03	9.84	8.74	8.68	9.14	Chr 14
ENST00000554532	AC005519.4	1.92	0.1	down	8.97	9.29	7.87	9.74	9.72	9.49	Chr 14
ENST00000557989	RP11-680F8.3	1.91	0.02	down	9	8.52	8.33	9.8	9.56	9.3	Chr 15
ENST00000413063	WASF3-AS1	1.89	0.03	up	9.64	10.17	9.29	8.69	8.96	8.68	Chr 13
ENST00000564455	AC009120.5	1.89	0.1	down	8.88	10.03	8.75	10.38	10.02	10.01	Chr 16
ENST00000544717	RP11-319E16.1	1.89	0.1	up	9.84	10.24	9.81	8.26	9.52	9.37	Chr 12
ENST00000579057	LINC00271	1.88	0.1	up	8.98	9.84	9.63	8.11	8.39	9.22	Chr 6
ENST00000455894	LINC00378	1.88	0.06	up	10.11	10.38	9.96	8.63	9.37	9.72	Chr 13
ENST00000513893	LINC00461	1.87	0.08	down	8.72	9.57	9.05	10.05	9.5	10.5	Chr 5
ENST00000537095	FZD10-AS1	1.86	0.03	up	9.98	10.42	9.91	9.08	8.91	9.62	Chr 12
ENST00000568707	CTD-2323K18.1	1.86	0.02	up	9.59	9.43	9.46	8.84	8.84	8.11	Chr 15

To maximize the coverage of lncRNA expression profiling, another two publicly available gene expression data, GSE55909 and TCGA, were also used to identify the differentially expressed lncRNAs in prostate cancer. We firstly analyzed GSE55909 database and identified 318 lncRNAs produced from 250 genes were up-regulated and 1927 lncRNAs produced from 1653 genes were down-regulated in prostate cancer compared with normal tissues. Hierarchical clustering of the differentially expressed lncRNAs was shown in Figure 1B. Then, RNAseq data from TCGA, a cohort of 52 normal prostate tissues and 419 prostate cancer samples, were analyzed. According to OE Human lncRNA Microarray V2.0, which contains 4321 lncRNAs with description from NCBI, there are 1410 lncRNAs that can be found in TCGA dataset. After filtering the dataset to remove the lncRNAs that have low expression levels, we ended up with 654 expressed lncRNAs that are potentially relevant in prostate cancer (Supplementary Table S3). Compared with the normal prostate tissues, we found that there were 252 lncRNAs that were up-regulated (*p* < 0.05) and there were 157 lncRNAs that were down-regulated (*p* < 0.05) in prostate cancer. Hierarchical clustering of the differentially expressed lncRNAs was shown in Figure 1C.

To identify androgen-responsive lncRNAs, Agilent Human lncRNA array was performed to simultaneously observe lncRNAs and mRNAs expressions in androgen-dependent LNCaP cells under DHT stimulation in time points of 0 h and 2 h, respectively. The ‘0 h’ worked as the control representing cellular status before DHT stimulation. Supervised analysis of the microarray data showed 3767 deregulated lncRNA transcripts (1991 transcripts up-regulated and 1776 transcripts down-regulated) produced from 2980 genes, with an average expression level over 2-fold change in 2 h compared to 0 h (GSE72866). The list of the top 15 upregulated and top 15 downregulated transcripts identified by microarray analysis was shown in Table 2.

**Table 2:** Top 30 differentially expressed transcripts under DHT stimulation identified by microarray analysis

ProbeName	Ensembl Transcript ID	Gene Symbol	FC (abs)	Regulation	0 hr	2 hr	Chr
oebiotech_09219	ENST00000419431	LINC00308	47.02	up	3.61	9.16	Chr 21
A_21_P0010982		MALAT1	41.79	up	8.17	13.55	Chr 11
A_21_P0014694		LOC100509487	41.35	up	3.37	8.74	Chr 16
oebiotech_21382	ENST00000569100	GOLGA8B	38.43	up	4.13	9.39	Chr 15
oebiotech_20175	ENST00000507787	HERC2P9	37.47	up	3.46	8.68	Chr 15
A_19_P00331618		LOC100507645	37.41	up	8.88	14.11	Chr 11
oebiotech_16752	ENST00000425771	GAS5	37.04	up	3.71	8.92	Chr 1
oebiotech_17693	ENST00000508832	MALAT1	35.05	up	9.77	14.9	Chr 11
A_33_P3291430	ENST00000397750	ST7-OT4	34.61	up	3.37	8.48	Chr 7
oebiotech_05345	ENST00000546959	LINC01296	34.44	up	3.68	8.78	Chr 14
oebiotech_20049	ENST00000429124	FTX	30.87	up	3.4	8.35	Chr X
A_21_P0011477	ENST00000440946	AP000525.10	29.83	up	3.74	8.64	Chr 14
A_21_P0010871	ENST00000445221	BMS1P1	29.65	up	4.8	9.69	Chr 10
A_21_P0011370	ENST00000360553	GOLGA8A	29.04	up	3.89	8.75	Chr 15
A_19_P00319476		LOC100507645	28.89	up	8.63	13.49	Chr 11
A_21_P0001069	ENST00000416908	RP11-449J1.1	32.29	down	8.45	3.44	Chr 1
oebiotech_00606	ENST00000419425	AC006369.2	12.13	down	8.02	4.42	Chr 2
A_21_P0002731	ENST00000425195	AC098973.2	11.22	down	7.07	3.58	Chr 3
A_21_P0001785		FLJ33534	10.82	down	6.85	3.41	Chr 2
A_21_P0011663		GTSCR1	10.06	down	7.52	4.19	Chr 18
oebiotech_02576	ENST00000455447	TNR-IT1	9.09	down	6.58	3.39	Chr 1
oebiotech_06568	ENST00000561479	RP11-586K12.4	8.85	down	6.73	3.58	Chr 16
oebiotech_03425	ENST00000507698	RP11-438D8.2	8.62	down	6.68	3.57	Chr 3
A_21_P0002701	ENST00000429949	LINC00690	8.53	down	6.46	3.37	Chr 3
A_21_P0014499		SACS-AS1	8.01	down	7.2	4.2	Chr 13
A_21_P0004212	ENST00000512521	RP11-121L11.3	7.53	down	7.5	4.59	Chr 5
A_19_P00316241	ENST00000504068	RP11-434D9.1	7.38	down	6.37	3.48	Chr 5
oebiotech_16555	ENST00000413238	LINC00689	7.17	down	6.33	3.49	Chr 7
oebiotech_17270	ENST00000460833	ADAMTS9-AS2	7.02	down	6.21	3.4	Chr 3
oebiotech_05235	ENST00000543848	RP11-7M8.2	6.84	down	6.55	3.78	Chr 12

Noteworthy, being androgen-responsive lncRNAs does not mean being directly regulated by activated AR. Our major aim was to pick out AR directly regulated lncRNAs, which may play important roles in mediating AR signaling and PCa androgen-resistance transformation. ChIPBase data [25] was used and showed 312 androgen-responsive lncRNAs (including 10 kb up- and 1 kb downstream region of lncRNAs' transcription start site) overlapping AR peaks (Supplementary Figure S1; Supplementary Tables S1, S2) may be directly regulated by AR.

To identify androgen-responsive lncRNAs as diagnostic and prognostic markers for prostate cancer, we performed integrated analysis of differentially expressed lncRNAs and androgen-responsive lncRNAs in PCa patients. A total of 44 androgen-responsive lncRNAs were found to be over-expressed and 95 lncRNAs were found to be down-regulated in PCa samples (Figure 1D and 1E). We are primarily focusing on the up-regulated lncRNAs as putative biomarkers in prostate tumor compared with normal tissues. Of 44 up-regulated androgen-responsive lncRNAs, 3 androgen-reduced lncRNAs and 7 androgen-induced lncRNAs were randomly selected for further study.

### Validation of androgen-responsive lncRNAs using qRT-PCR

**Figure 2:**
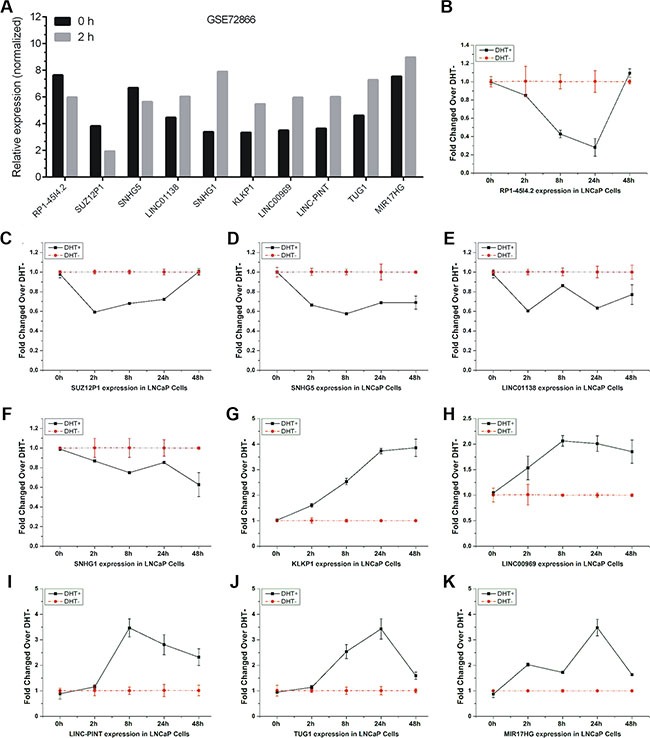
Validation of androgen-responsive lncRNAs in a time-dependent study The expression level of candidate lncRNAs from Agilent microarray data were shown (**A**). (**B**–**K**) RT-PCR analyses of ten lncRNAs' expressions in LNCaP cells treated with 10 nM DHT in time series of 0 h, 2 h, 8 h, 24 h and 48 h. Values of expressions treated with equal volume of vehicle in the same time series were used as control. Results are presented as the means ± s.d. of three independent experiments.

To further confirm the time-course microarray data, 10 lncRNAs (RP1-4514.2, SUZ12P1, SNHG5, LINC01138, SNHG1, KLKP1, LINC00969, LINC-PINT, TUG1 and MIR17HG) were selected for further qRT-PCR validation. The expression level of these lncRNAs from Agilent microarray data were shown in Figure 2A.

We firstly performed qRT-PCR analysis to observe lncRNAs expressions in LNCaP cells under DHT stimulation in a time series of 0 h, 2 h, 8 h, 24 h and 48 h, respectively. Five lncRNAs, RP1-4514.2, SUZ12P1, SNHG5, LINC01138, and SNHG1, were down-regulated under DHT treatment (Figure 2B–2F). Five lncRNAs, KLKP1, LINC00969, LINC-PINT, TUG1 and MIR17HG, were up-regulated under DHT treatment (Figure 2G–2K). Notably, RP1-4514.2 and LINC01138 were induced and KLKP1 was reduced over 2 fold after AR silenced, suggesting the involvement of AR in androgen-mediated regulation of these lncRNAs expressions. Puzzlingly, these results are not completely consistent with our microarray data.

**Figure 3:**
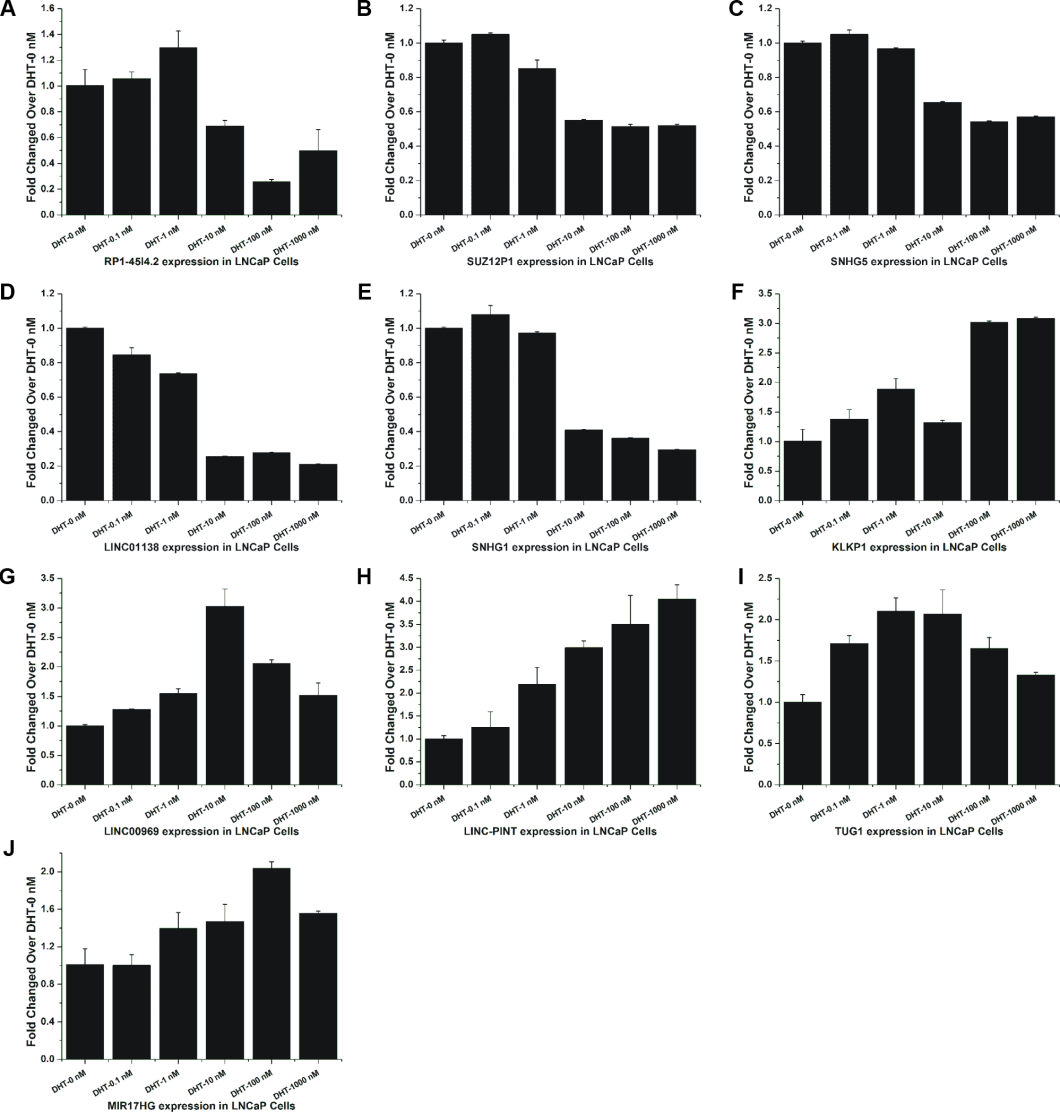
Validation of androgen-responsive lncRNAs in a dose-dependent study (**A**–**J**) RT-PCR analyses of ten lncRNAs' expressions in LNCaP cells treated with DHT for 24 h in dose series of 0 nM, 0.1 nM, 1 nM, 10 nM, 100 nM and 1000 nM. Values of dose zero were used as control. Results are presented as the means ± s.d. of three independent experiments.

Considering the limited time points and high false-positive rate of microarray, a dose-response study was recruited to explore the expression of these lncRNAs under different doses of DHT (0, 0.1, 1, 10, 100 and 1000 nM). The results showed that these lncRNAs had the same expression tendency under DHT stimulation in a time series and in a dose series (Figure 3A–3J). In addition, the expression of LINC00308, the most up-regulated lncRNA in our data, in LNCaP cells treated with DHT in dose or time dependent study were also analyzed by qRT-PCR (Supplementary Figure S3A–S3B). The expression of androgen-responsive genes PSA (KLK3) and TMPRSS2 were used as positive controls (Supplementary Figure S2A–S2D).

### Verification the regulation of AR on androgen-responsive lncRNAs

**Figure 4:**
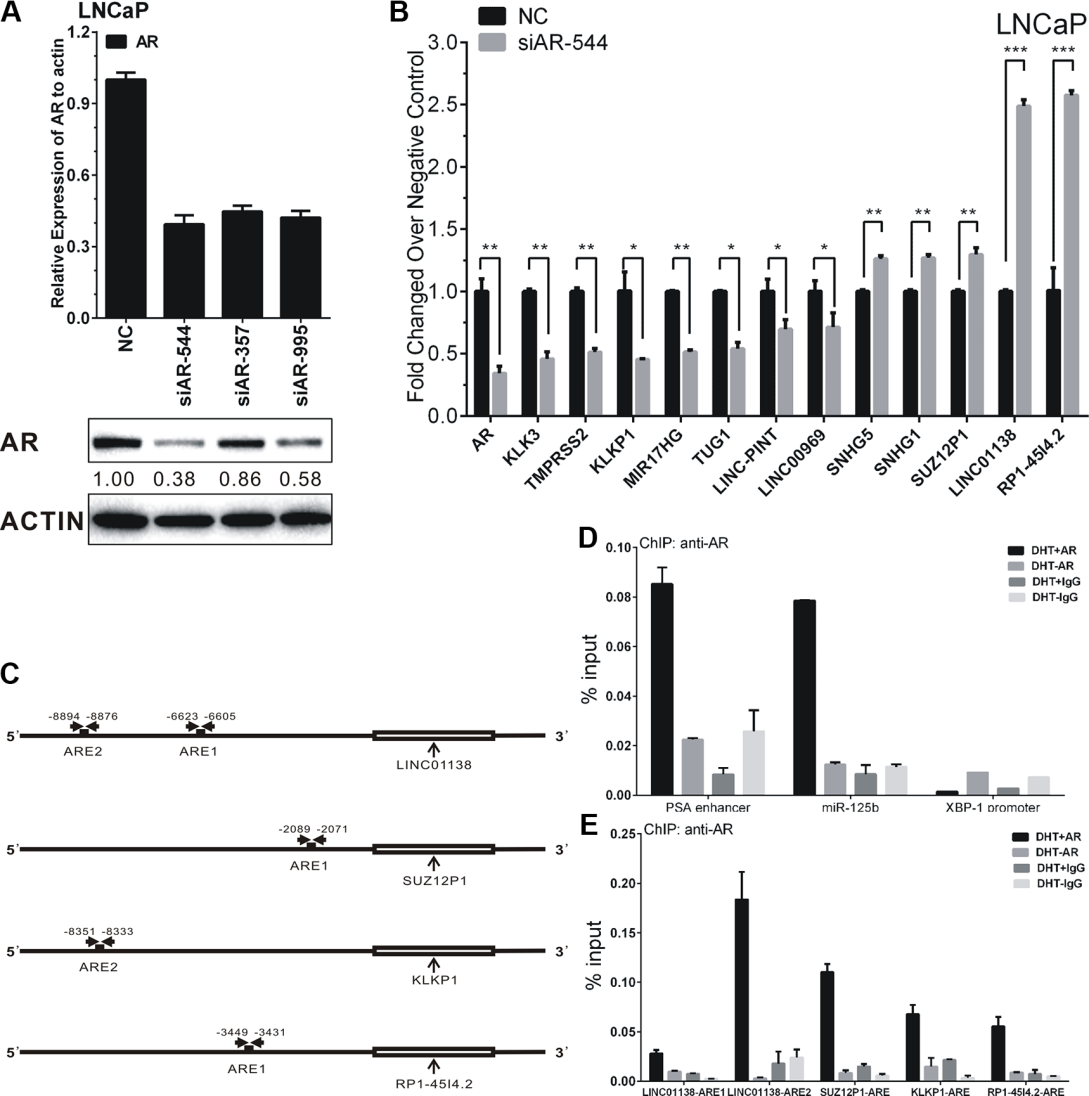
AR's regulation on lncRNAs' expression in LNCaP cells (**A**) The efficiency of siAR-544, siAR-357 and siAR-995 effecting on AR expression were confirmed by RT-PCR and western blot. Protein bands from Western blot assay were quantified using Quantity One software (Bio-Rad, USA). (**B**) Expressions of ten lncRNAs after transfection of siAR-544 compared with NC in LNCaP cells. (**C**) Schematic diagram of AREs of LINC01138, SUZ12P1, KLKP1 and RP1-45I4.2. The approximate ARE locations were indicated by the horizontal arrows. (**D**–**E**) ChIP assay of AR-binding on candidate AREs of LINC01138, SUZ12P1, KLKP1 and RP1-45I4.2. ChIP assays of PSA enhancer (KLK3) and miR-125b ARE serve as positive control, and XBP-1 promoter serve as negative control for AR-binding. Values, expressed as percentages of input DNA, are presented as the mean ± SD of at least three independent experiments. Significance was defined as *p* < 0.05 (*p < 0.05; **p < 0.01; ***p < 0.001).

To further validate the regulation of AR on lncRNAs, we performed siRNA-directed knockdown of AR in LNCaP cells. As shown in Figure 4A, siAR-544 remarkably decreased AR both mRNA and protein levels in LNCaP after siRNA transfection.

As our results shown, the expressions of five androgen-reduced lncRNAs, RP1-4514.2, SUZ12P1, SNHG5, LINC01138, and SNHG1, were significantly up-regulated after AR knockdown, the expressions of five androgen-induced lncRNAs, KLKP1, LINC00969, LINC-PINT, TUG1 and MIR17HG, were significantly down-regulated after AR knockdown (Figure 4B). The expression of androgen-responsive genes PSA (KLK3) and TMPRSS2 were used as positive controls.

RP1-4514.2, LINC01138, and SUZ12P1, the top three up-regulated lncRNAs, and KLKP1, the most down-regulated lncRNA after AR knockdown, were selected to validate the direct binding of AR to androgen response elements (AREs). The Genomatix database [26] was employed to predict potential androgen response elements (AREs) in the upstream 10 kb of the four lncRNAs transcription start site (TSS) (Figure 4C). AREs with ‘Core Similarity = 1′, which represent the highest match between target DNA sequence and ARE's conserved bases, were chosen for further validation by ChIP. As the results, compared with control, androgen-activated AR was significantly recruited to the predicted AREs of RP1-4514.2, LINC01138, SUZ12P1, and KLKP1, in the presence of 100 nM DHT in LNCaP cells for 4 h (Figure 4E). PSA enhancer (KLK3 promoter) and miR-125 b AREs were serves as the positive control for AR-binding, and XBP-1 promoter were serves as the negative control (Figure 4D). Collectively, all four lncRNAs are demonstrated as directly AR-targeted lncRNAs in androgen-dependent PCa.

### Validation of differentially expressed lncRNAs in patient samples and PCa cell lines using qRT-PCR

**Figure 5:**
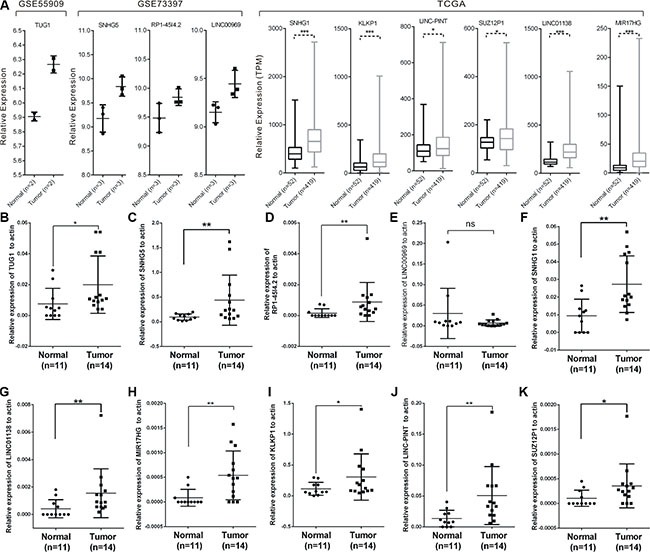
Expressions of candidate lncRNAs in tissue samples The expression level of candidate lncRNAs from microarray data and TCGA data were shown (**A**). (**B**–**K**) The expression levels of ten lncRNAs in 11 normal prostate tissues and 14 prostate cancer tissues measured by RT-PCR. Significance was defined as *p* < 0.05 (*p < 0.05; **p < 0.01; ***p < 0.001).

We then validated the expression of differentially expressed lncRNAs in PCa patients to investigate their clinical value. The expressions of these ten lncRNAs in their corresponding databases were presented in Figure 5A. To further validate the results, we examined the expression levels in 11 normal prostate tissues and 14 prostate cancer tissues with real-time PCR (Figure 5B–5K). All of these lncRNAs except LINC00969, were expressed significantly higher in prostate cancer samples compared with normal prostate tissues, which were consistent with the TCGA and microarray results.

**Figure 6:**
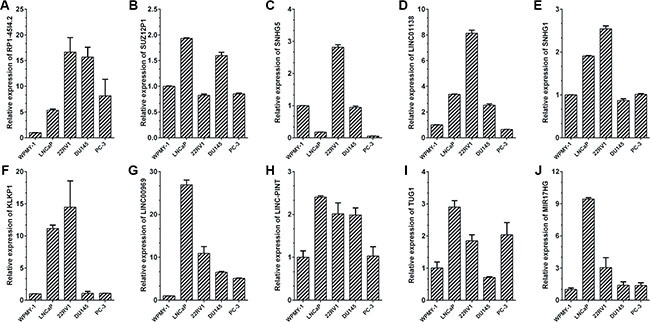
Expressions of candidate lncRNAs in prostate cancer cell lines qRT-PCR analysis of ten lncRNAs' expression levels in normal prostate epithelial cells line WPMY-1 and four prostate cancer cell lines, including LNCaP, 22Rv1, DU145 and PC-3. The Ct value of each lncRNA was normalized to the Ct value of β-actin, and the relative expression was calculated by comparing with WPMY-1 cells by calculating the 2^−ΔΔCt^ method. Each sample was run in triplicate to ensure quantitative accuracy.

Then, the expression levels of ten lncRNAs were validated in the noncancerous prostatic cells WPMY-1 and four human prostate cancer cell lines 22RV1, DU145, PC-3 and LNCaP by qRT-PCR (Figure 6). It turned out that most of the ten lncRNAs were up-regulated in prostate cancer cell lines compared with WMPY-1 and showed a similar expression pattern in patient samples. In addition, the expression of CCND2-AS2, the most up-regulated lncRNA in our data, in tissue samples and prostate cancer cell lines were also analyzed by qRT-PCR (Supplementary Figure S3C–S3D).

### Androgen-responsive lncRNAs can serve as diagnostic markers in patient tissues

**Figure 7:**
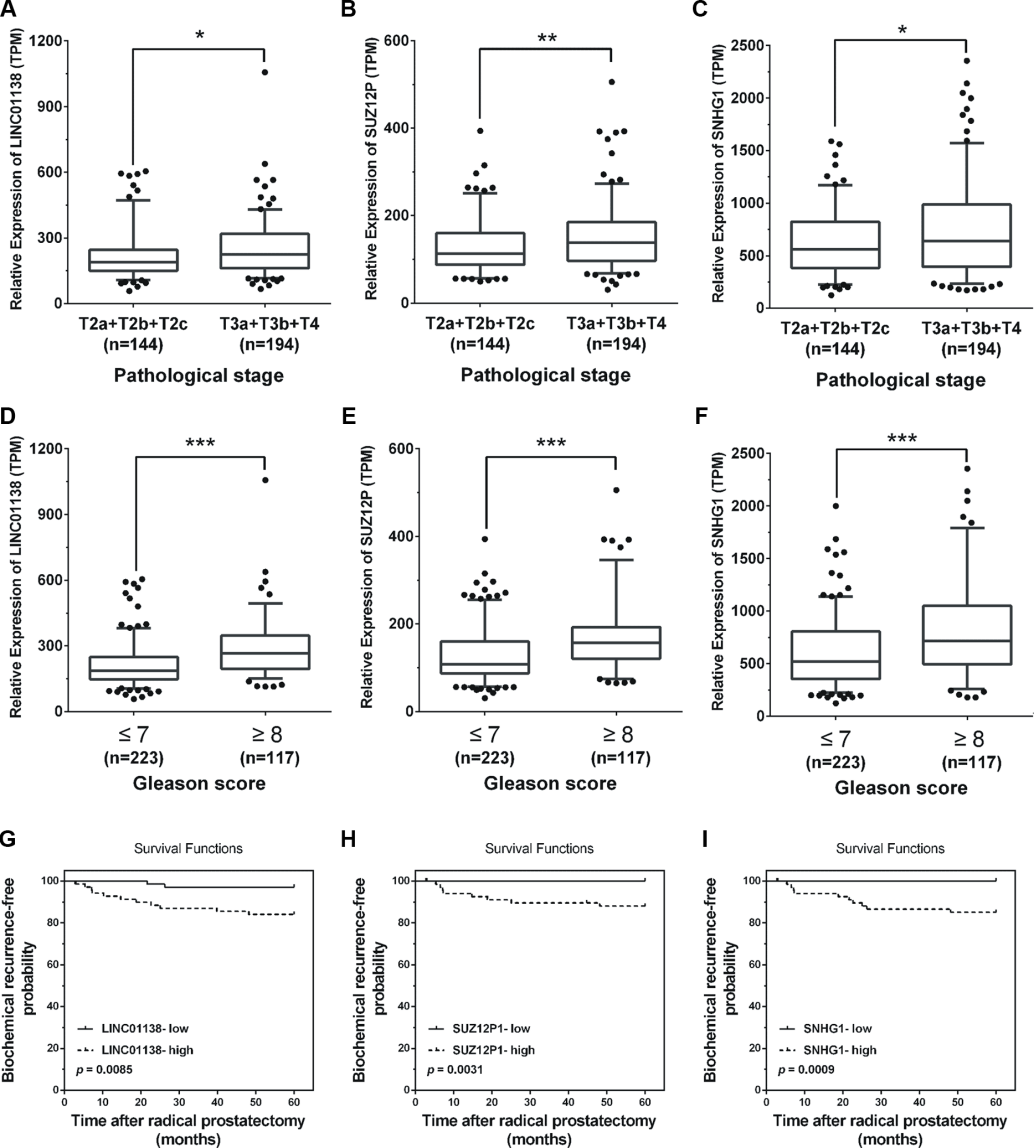
Pathological analyses of LINC01138, SUZ12P1 and SNHG1 (**A**–**C**) High expression levels of LINC01138, SUZ12P1 and SNHG1 in invasive extraprostatic tumors (pT3a, pT3b and T4 stages) compared with intraprostatic localized tumors (pT2a, pT2b and pT2c stages) according to publicly available gene expression data TCGA. (**D**–**F**) Low expression levels of LINC01138, SUZ12P1 and SNHG1 expression levels in prostate tumors with a low Gleason score (≤ 7) compared with tumors with Gleason score ≥ 8 according to publicly available gene expression data TCGA. (**G**–**I**) Kaplan-Meier curves for survival time after radical prostatectomy in patients with prostate cancer according to expression of LINC01138, SUZ12P1 and SNHG1. Significance was defined as *p* < 0.05 (*p < 0.05; **p < 0.01; ***p < 0.001).

As shown in Figure 1E, a total of 24 androgen-responsive lncRNAs were found to be up-regulated in TCGA. To evaluate possible prognostic value of these lncRNAs, we conducted a comprehensive bioinformatic analysis of TCGA database to identify the relationship between the expression of 24 lncRNAs and pathological grading and observed 3 androgen-responsive lncRNAs showed significant association with prostate cancer progression (Supplementary Tables S4, S5). As shown in Figure 7A–7F, LINC01138, SUZ12P1 and SNHG1 were expressed at lower levels with a low Gleason score (≤ 7), compared with tumors with Gleason score ≥ 8. Furthermore, the expression level of LINC01138, SUZ12P1 and SNHG1 markedly increased in invasive extraprostatic tumors (pT3a , pT3b and T4 stages) as compared with intraprostatic localized tumors (pT2a , pT2b and pT2c stages).

Next, we used a Kaplan-Meier analysis to evaluate whether 24 androgen-responsive lncRNAs expression were associated with patient outcome and 11 lncRNAs were found to be (*p* < 0.05). As expected, we found that a high level of LINC01138 (*p* = 0.0085), SUZ12P1 (*p* = 0.0031) and SNHG1 (*p* = 0.0009) were associated with significantly lower biochemical recurrence (Figure 7G–7I), indicating that the high level of LINC01138, SUZ12P1 and SNHG1 were correlated with a short biochemical recurrence-free survival times.

### LINC01138 and SUZ12P1 promoted the proliferation and inhibited apoptosis of PCa

**Figure 8:**
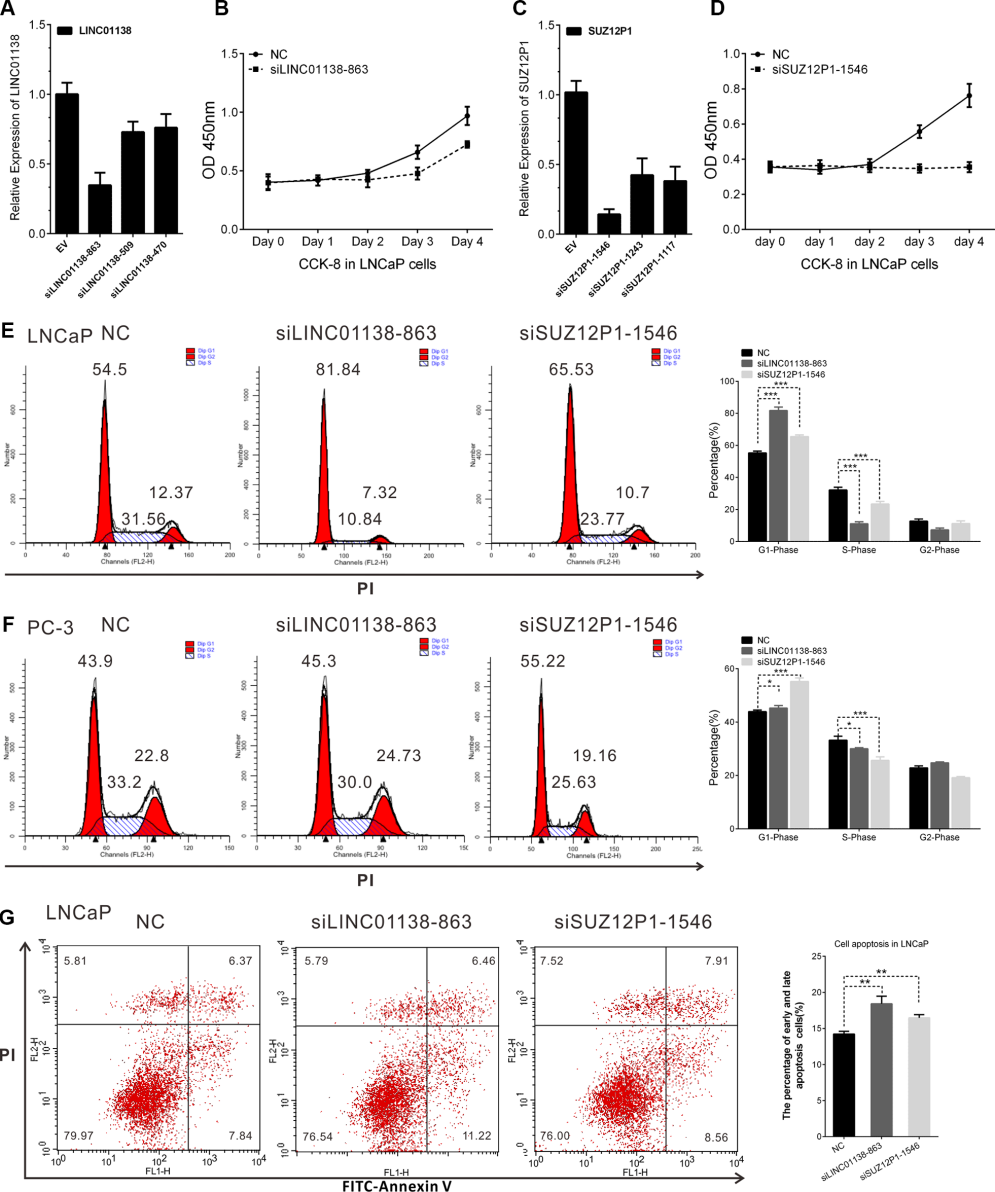
LINC01138 and SUZ12P1 promotes cell proliferation, cell cycle and inhibits apoptosis of PCa (**A** and **C**) The efficiency of siRNAs effecting on LINC01138 and SUZ12P1 expression were confirmed by RT-PCR. (**B** and **D**) LINC01138 and SUZ12P1 knockdown significantly inhibit LNCaP cell proliferation. (**E**, **F**) Cell cycle assay was performed in LNCaP and PC-3 cells. Cells were transfected with siLINC01138 or siSUZ12P1 for 48 h, stained with PI and evaluated with a FACScalibur flow cytometer. LINC01138 and SUZ12P1 knockdown inhibit cell cycle progression in LNCaP and PC-3 cells. (**G**) Cell apoptosis assay was performed with flow cytometer. Cells were transfected with siLINC01138 or siSUZ12P1 for 48 h, and subjected to cell apoptosis assay. LINC01138 and SUZ12P1 knockdown in LNCaP cells increased the fraction of both early apoptotic cells and late apoptotic cells. The cell cycle and apoptosis analysis results presented as mean ± SD (*n* = 3). Significance was defined as *p* < 0.05 (*p < 0.05; **p < 0.01; ***p < 0.001); ns means not significant.

To evaluate the biological functions of these lncRNAs, we picked two lncRNAs, LINC01138 and SUZ12P1, and used small interfering RNA (siRNA) to down-regulate their expression in LNCaP cells. The CCK8 assay showed that knockdown of LINC01138 or SUZ12P1 markedly suppressed cell viability in LNCaP cells (Figure 8A–8D and Supplementary Figure S4A–S4B). We also observed that LINC01138 or SUZ12P1 knockdown in LNCaP and PC-3 cells significantly increased the proportion of cells in G1 phase and decreased that of cells in S phase by flow cytometry (Figure 8E–8F and Supplementary Figure S4C–S4F). We then explored the influence of LINC01138 and SUZ12P1 on apoptosis of prostate cancer cells lines using cell apoptosis assay, and found knockdown of them increased the fraction of apoptotic cells in LNCaP cells lines (Figure 8G). The results revealed that LINC01138 and SUZ12P1 functioned as oncogenes in prostate cancer.

## DISCUSSION

The molecular mechanisms involved in the development and androgen-resistance transformation of PCa remains unclear. Therefore, further study of castration-resistant PCa is of great importance. Recent studies indicate that lncRNAs play an important role in tumorigenesis and tumor metastasis [27–29]. In previous studies, several lncRNAs , including CTBP1-AS [6], PCAT18 [16], and PCAT29 [17], have been identified as targets of AR and were associated with PCa androgen-resistance transformation. Interestingly, NEAT1, an oestrogen receptor alpha-regulated lncRNA, promoted prostate tumorigenesis and was associated with therapeutic resistance as well [30].

In this study, we performed the dynamic microarray experiment for simultaneously observing expressions of genome-wide lncRNAs in LNCaP cells stimulated by DHT for 0 h and 2 h, respectively. Supervised analysis of the microarray data showed a total of 3767 lncRNAs (1991 up-regulated and 1776 down-regulated) produced from 2980 genes were differentially expressed after DHT stimulation. Combined with ChIPBase data [25], 312 lncRNAs overlapping AR peaks may be directly regulated by AR. As far as we know, this is the first study to identify androgen-responsive lncRNAs globally.

Another important topic is the exploration of prognostic markers for PCa, especially for castration-resistant PCa. To identify novel diagnostic and prognostic markers, we firstly investigated lncRNAs expression signature of PCa samples from patients. Another two publicly available gene expression data, TCGA and GSE55909 were also used to identify the differentially expressed lncRNAs in prostate cancer. Three thousand and eighty-two lncRNA transcripts (934 up-regulated and 2148 down-regulated lncRNAs) were found to be significantly differentially expressed. Some studies showed that RNA-Seq methods (TCGA) and microarray-based methods did not have high coincidence degree while they both are good technologies to measure gene expression level [31]. In present study, only 12% of up-regulated and lncRNAs of TCGA data (31/252) were also identified to be overexpressed in microarray data, consistent with previous reports. To maximize the coverage of lncRNA expression profiling and hold more useful information for further study, we used the union of TCGA and microarray results instead overlap of TCGA and microarray results. Novel lncRNAs expression signatures were revealed in these tissues.

A total of 44 androgen-responsive lncRNAs were found to be over-expressed and 95 lncRNAs were found to be down-regulated in PCa samples (Figure 1D and 1E). Of 44 up-regulated androgen-responsive lncRNAs, 3 androgen-reduced lncRNAs and 7 androgen-induced lncRNAs were randomly selected for further study. Using qRT-PCR assay, we validated that RP1-4514.2, SUZ12P1, SNHG5, LINC01138, and SNHG1, were down-regulated, and KLKP1, LINC00969, LINC-PINT, TUG1 and MIR17HG, were up-regulated after DHT stimulation in both time- and dose-dependent manner. Furthermore, we found AR knockdown could promote expressions of androgen- reduced lncRNAs and inhibit androgen- induced lncRNAs' expression. Based on these results, we identified AREs of RP1-4514.2, LINC01138, SUZ12P1 and KLKP1 using ChIP assay. As a result, a significant increase of AR binding to the chromatin of putative AREs in RP1-4514.2, LINC01138, SUZ12P1 and KLKP1, was showed in LNCaP cells treated with 100 nM DHT for 4h. Moreover, qRT-PCR assay showed that these ten lncRNAs were significantly up-regulated in PCa tumors and cell lines.

To evaluated prognostic values of differentially expressed androgen-responsive lncRNAs in PCa ,we then analyzed TCGA database and found that more than 90 transcripts were significantly differentially expressed in invasive extraprostatic tumors (pT3a , pT3b and T4 stages) compared with intraprostatic localized tumors (pT2a , pT2b and pT2c stages). Our study identified three androgen-responsive lncRNAs, LINC01138, SUZ12P1 and SNHG1 were significantly over-expressed in more metastasis and higher tumor stage patients. Furthermore, we conducted a comprehensive analysis to identify the correlation between androgen-responsive lncRNAs levels and biochemical recurrence, and found that a high level of LINC01138, SUZ12P1 and SNHG1 expression was associated with high biochemical recurrence. Moreover, we found that knockdown of LINC01138 and SUZ12P1 significantly inhibited cell proliferation and promoted cell apoptosis. These results suggested that these lncRNAs may act as oncogenes. Puzzlingly, all of the three lncRNAs were androgen-reduced genes, indicating that the expression of LINC01138, SUZ12P1 and SNHG1 may be repressed by AR activity in “androgen-dependent” tumors. There may be other pathways regulating these lncRNAs expression in PCa.

Several limitations of this study should be noted. First, the time points of time-course microarray is limited, and that make it restrict to observe dynamic expression of lncRNAs. Second, due to the limited sample size of data in the present study, further studies based on larger series of patients are needed to confirm the significance of the signature. Last, additional function investigations of these lncRNAs on PCa are still needed.

In conclusion, our study represents the comprehensive analysis of androgen-responsive lncRNAs in prostate cancer for the first time. Analyzing the expression of these lncRNAs is likely to provide novel therapeutic targets for PCa, counting the general of the recently approved therapies for mCRPC targeting the AR pathway [16, 32]. We also analyzed the expression patterns of lncRNAs in PCa samples, and identified a set of lncRNAs that are aberrantly expressed between prostate cancers and normal controls as well as the aberrant expressed lncRNAs between invasive extraprostatic tumors and intraprostatic localized tumors. These data suggest that these lncRNAs may be used as potential prognostic targets and predict patient outcome. We believe this study provides useful information for exploring potential therapeutic and prognostic targets for prostate cancer, especially for castration-resistant PCa.

## MATERIALS AND METHODS

### Cell culture and androgen treatment

LNCaP cells were purchased from the American Type Culture Collection (Manassas, USA) which was confirmed by short tandem repeat (STR) analysis. 22RV1, DU145, PC-3 and WPMY-1 were obtained from Cell Bank of Chinese Academy of Sciences (Shanghai, China) where they were authenticated by mycoplasma detection, DNA-Fingerprinting, isozyme detection and cell vitality detection. All experiments were carried out by each cell line at passages below 30. The four prostate cancer cell lines were maintained in RPMI 1640 medium (Corning, USA) supplemented with 10% FBS (Hyclone, USA) and WPMY-1 in DMEM medium (Corning, USA) with 10% FBS, and they all cultured at 37°C in 5% CO2.

Androgen treatment assay was performed as described previously [33]. Briefly, LNCaP cells were cultivated in Phenol Red-free RPMI 1640 (GIBCO/BRL) supplemented with 10% charcoal-dextran-stripped FBS for 3 days before androgen treatment, then were induced with DHT at concentration of 10 nM. The genome-wide dynamic response to DHT was analyzed at five time points -0 h, 2 h, 8 h, 24 h and 48 h, where ‘0 h’ represents the state before androgen action.

### Tissue collection

The trial was approved by the Research Ethics Committee of Tongji Hospital and verbal consent was obtained from all patients. Three tumor tissues and three adjacent normal tissues were used for microarray analysis of lncRNAs. Fourteen tumor tissues and 11 adjacent normal tissues were used for an extra evaluation by qRT-PCR. All samples were collected from Tongji Hospital, a subsidiary of Shanghai Tongji University, between January 2001 and December 2013. The prostate cancer patients whom the tissues were obtained from underwent radical prostatectomy and did not receive any pre-operation treatment. The histopathological features of tumor specimens were classified according to the Gleason score system and 2002 TNM classification system.

### Microarray and expression data sets

Total RNA of samples was isolated by using TRIzol (Invitrogen) and the RNeasy mini kit (QIAGEN). Total RNA was quantified by the NanoDrop ND-2000 (Thermo Scientific) and the RNA integrity was assessed using Agilent Bioanalyzer 2100 (Agilent Technologies).

Total RNA from LNCaP cells, treated with 10 nM DHT for 0 and 2 hr, were hybridized to Agilent Human lncRNA (4*180K, Design ID: 042818). The sample labeling, microarray hybridization and washing were performed based on the manufacturer's standard protocols. Briefly, total RNA were transcribed to double strand cDNA, then synthesized into cRNA and labeled with Cyanine-3-CTP. The labeled cRNAs were hybridized onto the microarray. After washing, the arrays were scanned by the Agilent Scanner G2505C (Agilent Technologies). Feature Extraction software (version10.7.1.1, Agilent Technologies) was used to analyze array images to get raw data. Genespring were employed to finish the basic analysis with the raw data. The raw microarray data were uploaded to the Gene Expression Omnibus public repository (http://www.ncbi.nlm.nih.gov/geo/query/acc.cgi?acc = GSE72866; Gene Expression Omnibus series no. GSE72866). To begin with, the raw data was normalized with the quantile algorithm. The probes that at least 1 conditions out of 2 conditions have flags in “P” were chosen for further data analysis. Differentially expressed lncRNAs were then identified through fold change. The threshold set for up- and down-regulated genes was a fold change > = 2.0.

Global expressions of lncRNAs in 3 PCa samples and 3 normal prostate tissues were examined microarray version A10312-90-2 (RiboBio), containing 41532 lncRNA probes and 33352 mRNA probes. The sample labeling, microarray hybridization and washing were performed based on the manufacturer's standard protocols. The raw microarray data were uploaded to the Gene Expression Omnibus public repository (http://www.ncbi.nlm.nih.gov/geo/; Gene Expression Omnibus series no. GSE73397). Raw data was normalized with the log2 scale. Two-class unpaired significance analysis of microarray (SAM) [34] was employed to filter significantly differentially expressed lncRNA between PCa and normal samples (Reference). Following 1000 permutations, LncRNAs were selected with discriminating parameter of *q* < 0.05.

In this paper, only lncRNAs with description from NCBI or Ensemble were selected for further study.

Another two publicly available gene expression data, TCGA Data Portal (https://tcga-data.nci.nih.gov/tcga/) and GSE55909 [35], were also used to identify the differentially expressed lncRNAs in Prostate Cancer.

### RNA interference and transient transfection

All siRNA oligonucleotides against AR and negative control (NC) were were purchased from GenePharma (Shanghai, China), and used at 50 nM concentration. All sequences of synthetic oligonucleotides are listed in Supplementary Table S6. Transfection was carried out with Lipofectamine 2000 Transfection Reagent (Life, USA) according to the manufacturer's procedure.

### Real-time reverse transcription PCR (qRT-PCR) analysis

qRT-PCR for lncRNAs and mRNAs was performed using AceQ qPCR SYBR Green Master Mix (Vazyme Biotech co., ltd) [36]. Primers used for qRT-PCR were listed in Supplementary Table S6. The Ct values were normalized using β-actin as internal control to estimate the different expression of genes. Relative mRNA expression was calculated using the 2^−ΔΔCt^ method. Each sample was run in triplicate to ensure quantitative accuracy.

### Chromatin immunoprecipitation (ChIP) assay

ChIP was performed as described previously [37]. Chromatin immunoprecipitates for proteins and methyl marks were amplified by quantitative PCR, normalized to input, and calculated as percentages of inputs. Fold enrichment levels indicate the fold changes over the negative control immunoglobulin G (IgG). The primers for qRT-PCR analysis of DNA fragments containing ARE were listed in Supplementary Table S6, particularly KLK3 (PSA) enhancer and miR-125b ARE work as the positive control, whereas XBP-1 promoter works as the negative control. A DNA region without a putative ARE also served as negative control.

### Cell proliferation assay

Cell proliferation analysis was performed with Cell Counting Kit-8 (CCK-8, Dojindo Laboratories, Kumamoto, Japan) in octuplicate according to the manufacturer's instructions. Briefly, cells of 5000 per well were seeded into 96-well plate, and examined at the time point of 0, 24, 48, 72, 96, and 120 h. At each time point, CCK-8 (10 μl) was added to the wells, and after an incubation of 2 h at 37°C, absorbance was measured at 450 nm with a Microplate Reader ELx808 (Bio-Tek, VT, USA).

### Cell cycle and apoptosis assay

Cells were harvested 48 h after transfection. For cycle assay, cells were incubated with 0.03% triton X-100 and propidium iodide (PI) (50 ng/mL) for 15 min; the percentages of cells in different phases of cell cycle were measured with a FACScalibur flow cytometer (BD, CA, USA) and analyzed with ModFit software (Verity Software House, ME, USA). For apoptosis assay, cells were assayed with FITC Annexin V Apoptosis Detection Kit (BD, CA, USA) and analyzed by flow cytometry.

### Western blotting analysis

Western blotting was performed as described previously using antibodies against AR (Millipore) and actin (Sigma) [36]. The protein concentration was determined by the BCA Protein Assay Kit (Novoprotein Scientific Inc., China), in accordance with the manufacturer's instructions. Signal intensity of Western blots was quantified by Quantity One Software (Bio-Rad, USA).

### Statistical analysis

The numerical data were presented as mean ± standard deviation (SD) of at least three determinations. Statistical comparisons between groups of normalized data were performed using *T*-test or Mann–Whitney *U*-test according to the test condition. A *p* < 0.05 was considered statistical significance with a 95% confidence level.

## SUPPLEMENTARY MATERIALS FIGURES AND TABLES














