# Supplementary material for: Identification of androgen-responsive lncRNAs as diagnostic and prognostic markers for prostate cancer
Source: Oncotarget. 2016 Aug 19;7(37):60503–18. doi: 10.18632/oncotarget.11391 (PMC5312399; doi:10.18632/oncotarget.11391)
Supplement: Supplementary file 1 [file oncotarget-07-60503-s001.pdf]

Identification of androgen-responsive lncRNAs as diagnostic and prognostic markers for prostate cancer

Supplementary Materials

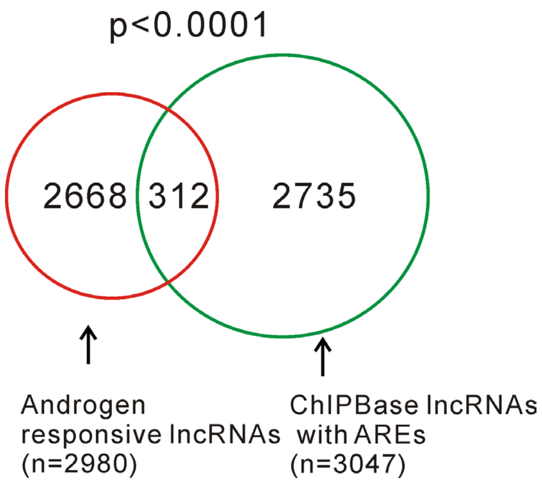

Supplementary Figure S1: Venn diagrams display the overlap of androgen responsive lncRNAs and ChIPBase lncRNAs with AREs.

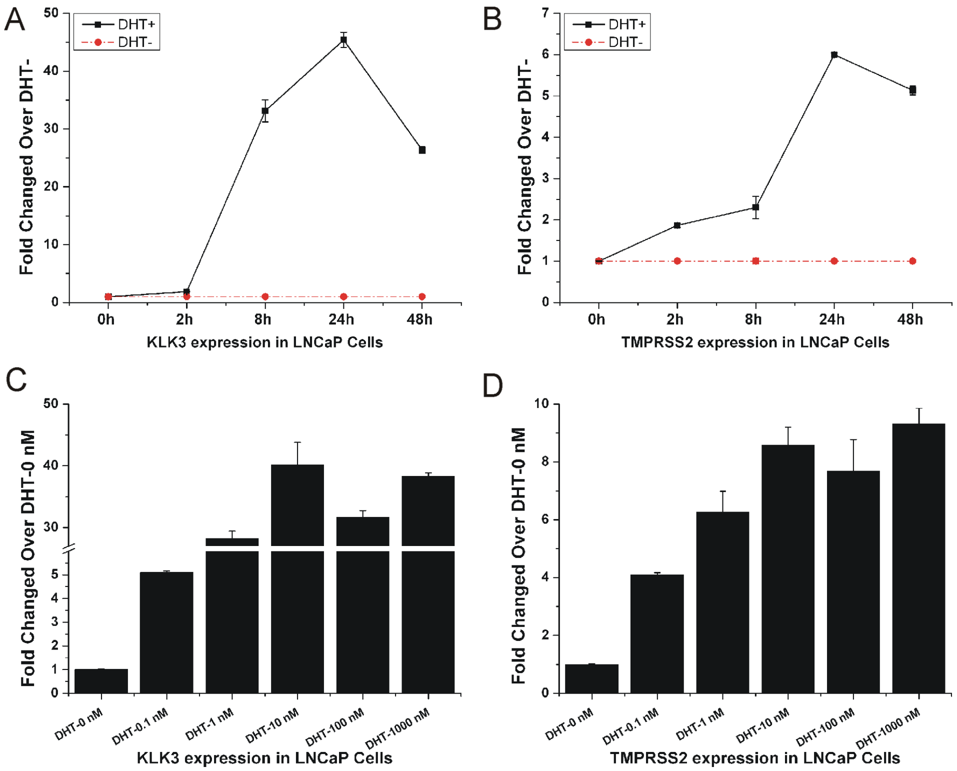

Supplementary Figure S2: The expression of androgen-responsive genes PSA (KLK3) and TMPRSS2 used as positive controls after DHT stimulation in LNCaP cells. Values are presented as the mean ± SD of at least three independent experiments.

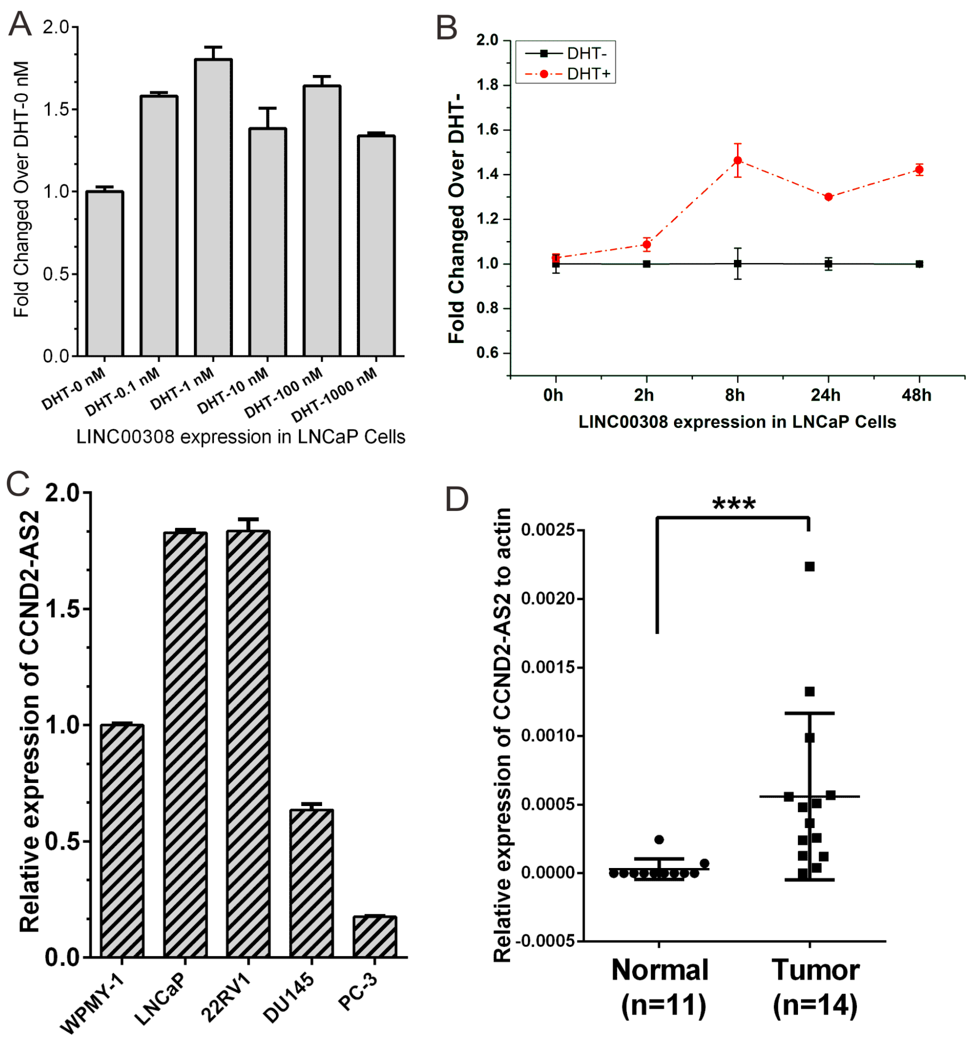

Supplementary Figure S3: (**A**–**B**) RT-PCR analyses of LINC00308 expression in LNCaP cells treated with DHT in a dose or time dependent study. (**C**–**D**) Expressions of CCNA2-AS2 in tissue samples and prostate cancer cell lines. Values of expressions treated with equal volume of vehicle in the same time series were used as control. Results are presented as the means ± s.d. of three independent experiments. Significance was defined as $p < 0.05$ ($*p < 0.05$; $**p < 0.01$; $***p < 0.001$).

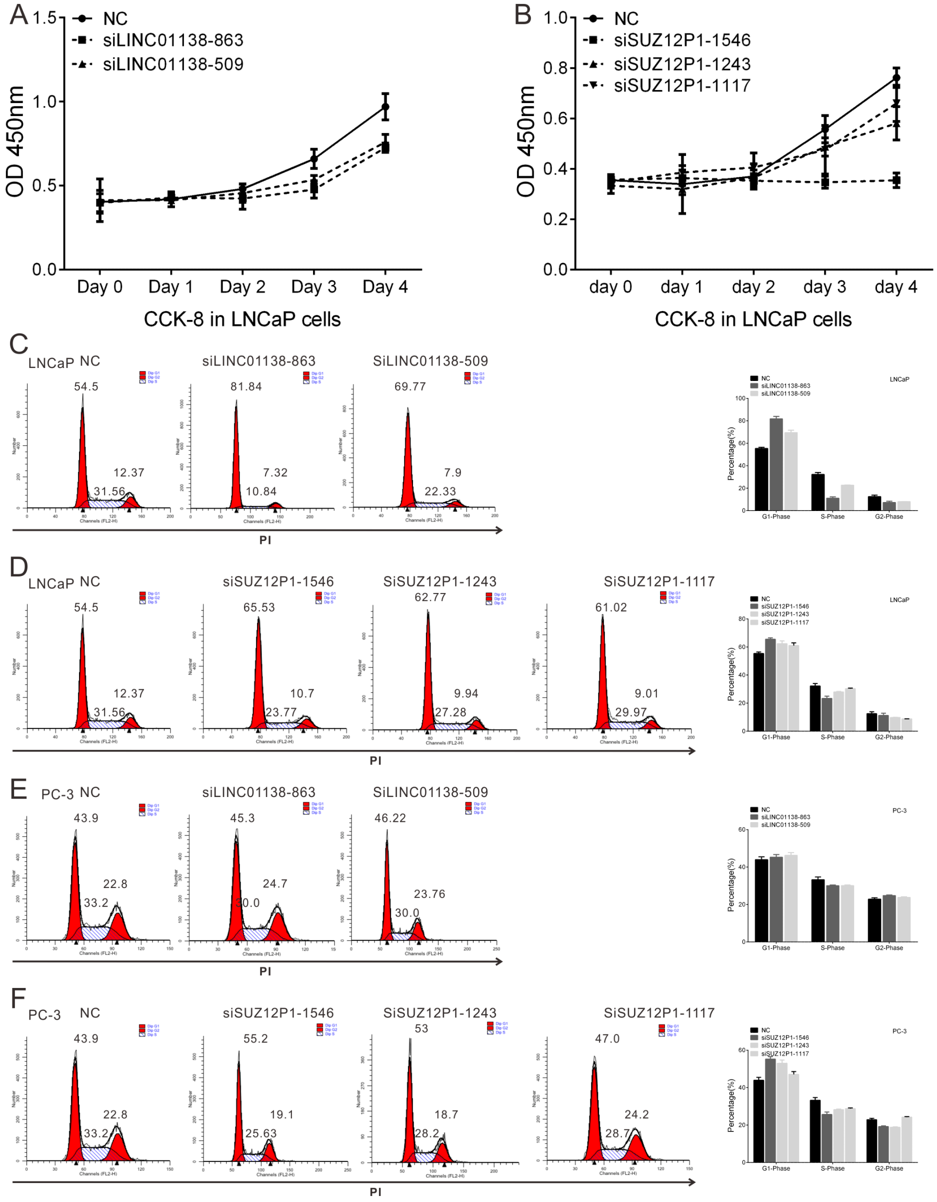

Supplementary Figure S4: LINC01138 and SUZ12P1 promotes cell proliferation, cell cycle and inhibits apoptosis of PCa. (**A**–**B**) LINC01138 and SUZ12P1 knockdown significantly inhibit LNCaP cell proliferation. (**C**–**F**) Cell cycle assay was performed in LNCaP and PC-3 cells. Cells were transfected with siLINC01138 or siSUZ12P1 for 48 h, stained with PI and evaluated with a FACS calibur flow cytometer. LINC01138 and SUZ12P1 knockdown inhibit cell cycle progression in LNCaP and PC-3 cells.

Supplementary Table S1: Results of lncRNAs (including 10 kb up- and 1 kb downstream region of lncRNAs' transcription start site) overlapping AR peaks based on ChIPBase data. See Supplementary Table_S1

Supplementary Table S2: Results of 451 androgen-responsive lncRNA transcripts (including 10 kb up- and 1 kb downstream region of lncRNAs' transcription start site) overlapping AR peaks based on ChIPBase data. See Supplementary Table_S2

Supplementary Table S3: Comprehensive analysis of differentially expressed lncRNAs (normal vs. tumor) in PCa patients based on TCGA dataset. See Supplementary Table_S3

Supplementary Table S4: Comprehensive analysis of differentially expressed lncRNAs (GleasonScore ≤ 7 vs. GleasonScore ≥ 8) in PCa patients based on TCGA dataset. See Supplementary Table_S4

Supplementary Table S5: Comprehensive analysis of differentially expressed lncRNAs (low pT-stage vs. high pT-stage) in PCa patients based on TCGA dataset. See Supplementary Table_S5

Supplementary Table S6: Primer sequences used for ChIP-PCR or qRT-PCR analysis and synthetic oligonucleotides sequences used transient transfection. See Supplementary Table_S6
